# Seeing More Than Human: Autism and Anthropomorphic Theory of Mind

**DOI:** 10.3389/fpsyg.2018.00528

**Published:** 2018-04-17

**Authors:** Gray Atherton, Liam Cross

**Affiliations:** ^1^Department of Psychological, Health and Learning Sciences, University of Houston, Houston, TX, United States; ^2^Department of Psychology, School of Science and Technology, Sunway University, Selangor, Malaysia; ^3^Department of Psychology, School of Science, University of Buckingham, Buckingham, United Kingdom

**Keywords:** anthropomorphism, autism, theory of mind, social cognition, perspective taking, mentalizing, animals

## Abstract

Theory of mind (ToM) is defined as the process of taking another’s perspective. Anthropomorphism can be seen as the extension of ToM to non-human entities. This review examines the literature concerning ToM and anthropomorphism in relation to individuals with Autism Spectrum Disorder (ASD), specifically addressing the questions of how and why those on the spectrum both show an increased interest for anthropomorphism and may even show improved ToM abilities when judging the mental states of anthropomorphic characters. This review highlights that while individuals with ASD traditionally show deficits on a wide range of ToM tests, such as recognizing facial emotions, such ToM deficits may be ameliorated if the stimuli presented is cartoon or animal-like rather than in human form. Individuals with ASD show a greater interest in anthropomorphic characters and process the features of these characters using methods typically reserved for human stimuli. Personal accounts of individuals with ASD also suggest they may identify more closely with animals than other humans. It is shown how the social motivations hypothesized to underlie the anthropomorphizing of non-human targets may lead those on the spectrum to seek social connections and therefore gain ToM experience and expertise amongst unlikely sources.

## Introduction

It took me a long time to figure out that I see things about animals other people don’t. And it wasn’t until I was in my forties that I finally realized I had one big advantage over the feedlot owners who were hiring me to manage their animals: being autistic. Autism made school and social life hard, but it made animals easy (Grandin and Johnson, 2009, p. 1).

Anthropomorphism is the ascription of human features to non-human entities (Epley et al., 2007), and it often occurs when non-human entities are perceived as behaving both intentionally and unpredictably (Waytz et al., 2010b). Perhaps one reason individuals are more likely to anthropomorphize entities that are unpredictable is that human behavior can be equally difficult to predict, governed by a complex system of non-observable cognitions, beliefs, and motivations (Evans and Stanovich, 2013). Luckily, early in life we learn to attend to nuances in behavior that allow for an intrinsic tracking of other’s intentions (Onishi and Baillargeon, 2005). Thus, when non-human entities behave invariably, we reflexively attempt to make sense of that behavior, by tracing it back to a particular goal or purpose.

The act of delineating a person’s goal or purpose involves using theory of mind (ToM). ToM is a form of social cognition that refers to the ascription and recognition of thoughts, emotions and beliefs to the self and others and the ability to recognize that another’s perspective is different from our own (Baron-Cohen, 1999). When people ponder the goals or motivations of non-human entities, they are essentially using ToM. Humanizing the behavior of non-human entities is a pathway toward using ToM to understand the entity’s motivations or intentions, thus anthropomorphism and ToM are closely connected (Epley et al., 2007). Areas of the brain such as the temporoparietal junction (TPJ), which activates in accordance with ToM, also activates when anthropomorphizing (Chaminade et al., 2007) and when rationalizing the behavior of both humans and animals (Spunt et al., 2017). Additionally, the more a person anthropomorphizes, the larger the areas of the brain that are responsible for ToM processing (Cullen et al., 2014), highlighting the connection between anthropomorphism and ToM.

There is some evidence that ToM and, by association, anthropomorphism, reflect a more general predictive strategy people use to process unpredictability in the environment, independent of any one agent’s human-like properties, called predictive encoding (Friston and Frith, 2015). For instance, in the “uncanny valley,” when a stimuli is presented as human, such as a humanoid robot, yet their behavior is *too* predictable or mechanical, numerous error signals are transmitted, and as a result it is difficult to predict the robot’s actions (Saygin et al., 2012). Thus, at its most basic level, it is likely that ToM, and in turn anthropomorphism, is triggered through a more general recognition of behavioral patterns through a process of predictive encoding.

However, it is also true that anthropomorphism is not simply the mind engaging in more general predictive strategies, but involves applying a specifically human schema to better understand non-human agents. This process can be observed when individuals take the Social Attribution Task, in which people increasingly attribute human behavioral patterns to animated shapes (Heider and Simmel, 1944). By humanizing non-human agents, individuals are better equipped to utilize familiar predictive encoding strategies. As people have extensive knowledge of the types of goals that underlie such behaviors in human agents, the more one humanizes for example an unpredictable gadget, the easier it becomes to predict the gadget’s future behavior (Waytz et al., 2010b). This helps explain why, in contrast, dehumanizing an agent, such as the robot in the “uncanny valley,” leads to particularly strong predictive encoding disruption (Saygin et al., 2012).

Arguably the largest store of knowledge about human agency comes from an understanding of one’s own behavioral antecedents and outcomes, which can aid in the representation of what may underlie a person’s actions. Humphrey (1984) refers to this as “reflexive consciousness” or the ability to map the externalizing behaviors of others onto the internal experience of the self. Evidence for reflexive consciousness within the brain has come through the discovery of a mirror neural network, which elicits the activation of one’s own motoric brain regions even when only passively viewing the actions of others(Kohler et al., 2002), as well as a “default network” in cortical midline structures of the brain, which activates in relation to both self-related and socially related thoughts (Uddin et al., 2007). Both networks reveal the important role self-conceptualization plays when both processing other’s actions and representing their mental states.

Therefore, it is likely that one reason people anthropomorphize is that they are not only “humanizing” an unfamiliar agent, but more specifically they are *personalizing* the agent to activate self-representations and simulate the other’s experience. Thus, it is not surprising that a critical effect following ToM and anthropomorphic engagement with another includes perceiving that agent as more similar to the self (Epley et al., 2007), in addition to viewing them more empathically (Waytz et al., 2010a), and displaying a greater desire to interact with them in socially desirable ways (Waytz et al., 2010a). As we develop expertise in using ToM to predict the actions of others, and even ourselves, we become most capable of understanding non-human agents by attributing human motivations to their behaviors, therefore giving rise to anthropomorphism (Waytz et al., 2010b). But what if a person does not develop such an interest and expertise in human cognition? What if they struggle to self-reference? Can they anthropomorphize?

Such questions are particularly pertinent with regard to autism spectrum disorder (ASD), a condition in which affected individuals show, in comparison to those who are neurotypical (NT), deficits in ToM (Baron-Cohen et al., 2015; Kana et al., 2015), poor self-referential cognition (Lombardo et al., 2007), decreased mirror neural activity (Oberman et al., 2005) and weakened connections within the default network (Weng et al., 2010), all of which are mechanisms conjectured to play an intrinsic role in anthropomorphizing. As will be explored throughout this review, despite these differences, which would presumably contribute to a particularly weakened ability to anthropomorphize, individuals with ASD appear to display an affinity for anthropomorphism and an even stronger performance on ToM tasks when agents are non-human. Explanations for relative strengths within this population in relation to the processing of anthropomorphic ToM will be discussed.

## Autism Spectrum Disorder

ASD is a neurodevelopmental disorder that affects approximately 1 in 68 individuals Christensen et al. (2016). Those affected possess atypical social and communicative styles, and restricted, repetitive behaviors and interests (American Psychiatric Association, [APA], 2013). Some believe that these two symptoms are somewhat separable (Brunsdon and Happé, 2014), as individuals with ASD often have significant variation in symptom profiles (Geschwind and Levitt, 2007). There are several prominent theories commonly used to explain the mechanisms believed to underpin ASD. Among them are the Empathizing/Systemizing theory (Baron-Cohen, 2009), the Enhanced Perceptual Functioning theory (Mottron et al., 2006), and the Social Motivation theory (Chevallier et al., 2012). These three theories largely center upon the hypothesized mechanisms which underlie the social and perceptual differences found in ASD, each will now be briefly explored.

The Empathizing/Systemizing theory of ASD is comprised of two elements; an empathetic/ToM deficit and a penchant toward systematic stimuli conforming to rule-based logic, such as numbers or mechanical objects (Baron-Cohen, 2009). Evidence of empathy deficits within ASD include cognitive difficulties such as failure to pass false belief tasks (Baron-Cohen et al., 1985), and affective impairments such as reduced ability to process facial emotions (Baron-Cohen et al., 2001), or poor automatic tracking of non-verbal cues (Schuwerk et al., 2015).

The systemizing element of the theory refers to the ability to understand and use rule-based reasoning or logic, which Baron-Cohen (2009) connects with an increased competence those with ASD often demonstrate in domains such as science and mathematics. However, as understanding social systems requires more “gestalt” or holistic interpretations, a penchant for systemizing may impede development in other areas. Indeed, research suggests that tendencies toward empathizing and systemizing have a strong inverse relationship in clinical samples (Grove et al., 2013), indicating that those with ASD may be approaching empathy tasks systematically. However, as will be highlighted in this review, several studies show those on the spectrum do not have a global deficit toward empathizing, as this theory suggests, and this ability is intact when social stimuli is anthropomorphic rather than human.

Enhanced Perceptual Functioning Theory (EPF) (Mottron et al., 2006) argues that people with ASD can indeed process globally, even at times showing strengths relative to controls (Perreault et al., 2011). However, it is hypothesized that the heightened perceptual sensitivities to lower order stimuli demonstrated by superior visual acuity (Gliga et al., 2015), sensitivity to musical pitch (Bonnel et al., 2003), motion perception (Foss-Feig et al., 2013) and even tactile sensitivity (for a review see Ben-Sasson et al., 2009), may lead to significant processing differences which may have downstream effects. For instance, as those with ASD show a diminished sensitivity to complex stimuli (Bertone et al., 2005; Boer et al., 2013) it may be that they increasingly rely on their enhanced lower-level sensory perception and thus struggle updating their processing strategies (Zaidel et al., 2015).

Heightened discriminatory abilities in relation to low-level object features in a domain (i.e., pitch, letters, digits or 2-D visuo-perceptual properties), may also underlie the circumscribed interests (CIs) in relation to a defined class of units often found to exist in this population (Baron-Cohen et al., 2009). CIs in ASD have been found to be particularly intense, interfering, and idiosyncratic compared to NTs (Anthony et al., 2013). The role of CIs in ASD with regards to processing advantages and disadvantages are themselves somewhat paradoxical in this population. For one, a person with ASD’s exposure to areas related to CIs can in many instances lead to “savant” type abilities in which a person shows extreme talent in relation to knowledge of a particular domain (Happé and Frith, 2010). However, research indicates that the presence of CI related stimuli can divert attention from social stimuli, and increase perseverative behaviors (Sasson et al., 2008). Some research indicates that in certain situations, such as when both a NT peer and child with ASD are interacting in relation to the child’s CI, such as playing with a toy boat or plane, social initiation is enhanced (Boyd et al., 2007). Children with ASD have also been shown to be more likely to follow another’s social gaze when directed toward CI stimuli (Thorup et al., 2017). This indicates that while CIs can divert social attention in this population, they can also be mechanisms for inducing positive social behaviors.

It has also been conjectured that the heightened sensory perception, and the presence of CIs, may carry a specifically social cost to those with the condition (Unruh et al., 2016). The Social Motivation Theory (SM) of ASD (Chevallier et al., 2012), argues that the population’s empathy and perceptual differences may arise through a reduced neurohormonal “reward” typically experienced when interacting socially with others (Chaminade et al., 2015a). Instead, stimuli representing restricted interests have been shown to activate reward circuitry usually stimulated through social contact (Grelotti et al., 2005; Foss-Feig et al., 2016).

While causality is difficult to infer, those that prescribe to a “social first” model of ASD believe that the enhanced ability to discriminate lower level stimuli may in part develop due to an absence of typical social development, such as the ability to engage in joint attention (Mundy et al., 2009). As young children with ASD are impaired in joint attention in the first years of life (Charman, 2003), and as joint attention is thought to underlie ToM (Sodian and Kristen-Antonow, 2015), it may be that lower level perceptual strengths develop in place of skills such as ToM which develop through more social learning methods.

Subsequent difficulties with skills like ToM have been shown to longitudinally impair social functioning and peer relations (Banerjee et al., 2011), and thus poor ToM may negatively influence a person with ASD’s motivation later in life to engage in social interactions. Research indicates that adults with ASD, have been shown to experience increased rates of loneliness, depression and anxiety, and cite social reasoning difficulties as a significant source of their isolation (Jobe and Williams White, 2007). Thus, an aspect of SM theory involves the possibility that decreased social reward processing may be in part a downstream consequence of the negative social experiences those with ASD symptoms often endure (Wood and Gadow, 2010).

Both the increased salience of lower level stimuli, particularly those that align with circumscribed interests (CI), and the decreased salience of non-systematic, social stimuli that may impact social motivation (SM), could help explain why people with ASD often have difficulties using ToM, which necessitates gestalt processing through complex modalities (for instance nonverbal body language coupled with explicit vocal communication), and socially directed attention (Frith and Frith, 2006). As ToM deficits have been shown to persist throughout development (Schneider et al., 2013) and correspond heavily to ASD symptom severity (Hoogenhout and Malcolm-Smith, 2017), it is an important mechanism for understanding ASD symptomology and trajectory. As research indicates that current ToM interventions demonstrate poor transfer into real life settings (Marraffa and Araba, 2016), finding ways in which ToM may be intrinsically rewarding to those with ASD, such as through anthropomorphism, could be a vital tool for researchers and community stakeholders alike. The ability and affinity to anthropomorphize in those with ASD will be explored in relation to the above theories throughout the remainder of this review.

## Anthropomorphism and ASD

There is some evidence that people with ASD, despite ToM deficits in relation to human stimuli, have intact or even enhanced ToM processing in relation to anthropomorphic stimuli, a claim which will be explored in more detail in subsequent sections. Theoretically, there are several reasons why such improvements may exist, and these will be discussed in connection with the three tenants of anthropomorphism from the Epley et al. (2007) model.

In the first tenet of the Epley et al. (2007) anthropomorphism model it is stated that individuals are more likely to anthropomorphize when they have an increased motivation for sociability. Support for this comes from research showing individuals with increased levels of loneliness are more likely to anthropomorphize pets (Epley et al., 2007), robots (Lee et al., 2006), and even smart phones (Wang, 2017). Research indicates that people with ASD are particularly vulnerable to loneliness and thus the anthropomorphizing of non-human agents may function as a social outlet of sorts. For instance, adults with a high degree of ASD related traits were found to be no different than controls in their desire for companionship, but reported significantly higher ratings of loneliness which they attributed to their lack of social understanding (Jobe and Williams White, 2007). Evidence of fewer social networks (Mazurek, 2014), along with an increased perception of the self as a poor social actor (Vickerstaff et al., 2007) may contribute to the elevated levels of social anxiety present within the population (for a review see MacNeil et al., 2009). As social differences may isolate those with ASD from peers and/or result in negative outcomes, anthropomorphizing non-human entities may allow for social engagement with less emotional risk. In this way, interactions with anthropomorphic characters may become more socially motivating, in line with SM theory.

In the second Epley et al. (2007) tenet, individuals are found to increasingly anthropomorphize a non-human entity to increase efficacy, and a desire for efficacy is heightened when the non-human’s behavior is increasingly unpredictable. One reason why individuals with ASD may increasingly anthropomorphize to increase efficacy is that properties of non-human creatures may map onto CIs, and are thus intrinsically rewarding to those with the condition (Dichter et al., 2010). Indeed, there have been several reported cases of children with ASD having restricted interests in relation to cartoons and animals (Grelotti et al., 2005; South et al., 2005; Turner-Brown et al., 2011), and in this way anthropomorphism may stem from a desire to increase efficacy in their restricted area of expertise. Additionally, the exaggerated physical appearance and motion of animals (Borgi and Cirulli, 2016) and cartoons (Rhodes et al., 1987) may heighten the perception of unpredictability in such agents, which leads to a desire for increased efficacy. Conversely, more nuanced behavioral cues indicating unpredictability when in human form, such as subtle changes in facial expression or gaze direction, may be more easily overlooked by those with ASD (Rump et al., 2009).

Lastly, in the third tenet, it is suggested that anthropomorphism is enhanced through the elicitation of agent knowledge, which often includes perceiving similarities between the self and the other. Individuals with ASD have been shown to have a diminished physical sense of self (Lombardo et al., 2010), and are also less sensitive to the physical irregularities of non-human agents (Kuriki et al., 2016; Kumazaki et al., 2017). Thus, it may be that a diminished physical sense of self allows individuals with ASD to view themselves in less human and more anthropomorphic ways, a viewpoint suggested in experiential accounts by those with the condition (Prince-Hughes, 2004). Thus, the increased social processing of anthropomorphic versus human agents in socially typical ways may reflect an elicitation of personal knowledge in relation to non-human entities through a processing of the self as “other than human” (Bergenmar et al., 2015).

To assess these claims, research investigating elements of social processing in individuals with ASD regarding human versus anthropomorphic stimuli will be explored. Processing of anthropomorphic versus human face and motion processing will first be discussed. Secondly, this review will explore how increased engagement with anthropomorphic stimuli can lead to ToM gains, along with a discussion of ASD interventions utilizing anthropomorphic engagement through animal and cartoon-based interventions. Finally, possible explanations for enhanced anthropomorphic interest, engagement, and social processing will be presented along with implications for practitioners and future research directions.

## Anthropomorphic Versus Human Face Processing

In this section, two one of the underlying mechanisms for understanding ToM, face processing and attention to eye gaze, will be examined. Aspects of face processing that differ in NTs and those with ASD are first discussed, along with the possible mechanisms driving these differences. Next, several studies are presented that demonstrate intact face processing in this population in relation to anthropomorphic characters, specifically cartoons, androids, and animals. Explanations for this differential processing are discussed along with implications for understanding ToM in this population.

## Typical Versus Atypical Face Processing

One of the integral components of ToM is conjecturing what a person is thinking by processing what their face is expressing (Baron-Cohen and Cross, 1992). It is thought that individuals begin to hone this ability immediately following birth, as infants are particularly interested in protofaces, or indistinct face-like shapes (Johnson et al., 1991), and can immediately mimic facial expressions (Meltzoff, 1999). However, prolonged exposure to familiar faces as “special” stimuli are likely responsible for the preference young children develop toward species-specific faces (Sugita, 2008), which in time develops into an expertise for species-specific facial recognition and facial emotion processing (Scherf et al., 2007).

Infants at risk for ASD have been shown to also orient toward faces, contrary to popular conceptions of ASD stemming from a nascent decreased social interest (Elsabbagh et al., 2013). Interestingly, de Klerk et al. (2014) found that at 7 months of age, infants at risk for ASD spent longer than is typical gazing at faces, yet surprisingly this was longitudinally linked to poorer facial recognition abilities. Thus, it was conjectured that the prolonged gazing at faces in infants at risk for ASD reflected piece-meal rather than holistic processing, meaning that rather than processing faces as “special” stimuli, they may have been processing them more in line with detailed objects. This may explain why NT children at age two have been found to be better able to differentiate human versus monkey faces, yet children with ASD do not develop this ability until 3–4 years of age (Chawarska and Volkmar, 2007). These differences suggest that while young children with ASD may gaze for longer at faces, they are not processing faces in a way that leads to typical facial recognition gains, which itself relies on holistic processing (Richler et al., 2011b). However, as will be discussed, it may be that those with ASD have developed an ability to process anthropomorphic faces in typical ways, which has implications for elucidating the social processing mechanisms in this population.

## Holistic Anthropomorphic Face Processing

It is conjectured that aspects of ToM depend on the ability to holistically process faces, allowing people to rapidly detect what may be a nuanced change in facial expression and to recognize familiar agents. To achieve this, individuals are thought to holistically compare a person’s face with a facial prototype, which allows for the distinct properties of a face to become salient (Farah et al., 1998). A significant body of research suggests that individuals with ASD show both qualitative and quantitative differences in the way they holistically process human faces (Tang et al., 2015). For instance, in a study by Pavlova et al. (2017), individuals with ASD were asked to process images of food which were arranged to look like faces. Unlike typically developed individuals, those with ASD showed significant difficulty recognizing that the food was arranged to look like a face, indicating a detailed, piece-meal interpretation of the stimuli.

One method for measuring holistic face processing is to measure the facial inversion effect, which refers to the significant difficulty NTs display when processing inverted rather than upright faces (Leder and Bruce, 2000), indicating disruption when a face does not conform to its typical configural pattern (Richler et al., 2011a). Research indicates that individuals with ASD show a decreased inversion effect when viewing human faces (Falck-Ytter, 2008; Senju et al., 2008; Vida et al., 2013). However, there are several studies indicating that individuals with ASD may demonstrate the inversion effect when faces are anthropomorphic, indicating that they are processing them holistically.

For instance, an investigation by Rosset et al. (2008) tested facial emotion recognition in children with ASD and NT controls using both cartoon drawings and human photographs of inverted and upright faces. They found that NT children showed the inversion effect for both cartoon and human faces, meaning that their holistic facial representations were significantly disrupted when both types of stimuli were inverted. However, individuals with ASD did not show this effect when viewing inverted human faces; instead, they demonstrated the inversion effect only when processing cartoon faces.

Interestingly, follow up research by Rosset et al. (2010) again tested the inversion effect in cartoon versus human faces, but this time participants were asked to discriminate facial features of stimuli. Results showed that NT participants demonstrated the inversion effect only when viewing human faces. In contrast, participants with ASD did not show a preference for either real or cartoon faces, performing equally in each condition, and showing a reduced inversion effect compared to controls. Together, these results illustrate a trend in which anthropomorphizing social stimuli can at times be advantageous for those with ASD. While anthropomorphism does not always lead to processing gains, as shown in Rosset et al. (2010), non-human presentation does not appear to interfere with ASD processing patterns.

As individuals with ASD have been shown to report a heightened engagement with cartoons (Kuo et al., 2014), it may be that the cartoon rather than human inversion effect reflects a greater degree of elicited agent knowledge in relation to this kind of stimuli. For instance, research indicates that the inversion effect is significantly strengthened when individuals view faces reflective of their own age and race (Ding et al., 2014), indicating that elicited agent knowledge enhances the anthropomorphism of similar facial stimuli. Additionally, the lack of inversion effect toward human faces may reflect a decreased anthropomorphizing of human faces, possibly due to a decreased ability to elicit agent knowledge in relation to humans. This is surprising, as individuals with ASD undoubtedly have significantly more experience with humans. However, as the Epley et al. (2007) model also posits, a desire for sociality interacts with the elicitation of agent knowledge. Thus, it may be that decreased salience for human faces, due to a possible social disengagement with human faces, does not interfere with cartoon processing. In the following section, research in ASD demonstrating intact processing of anthropomorphic rather than human faces will be discussed in relation to neural evidence.

## Fusiform Face Area (FFA)

One mechanism implicated in the holistic processing of faces is an acquired activation in the fusiform face area (FFA) when viewing facial stimuli. The FFA is a brain region located in the right hemisphere, where “holistic” processing is thought to occur, and this region is notably activated when NT individuals view faces (Carlei et al., 2017). However, as shown in research testing individuals with particular areas of expertise, it can also activate when a person views various non-face stimuli of significant personal interest and experience (Tarr and Gauthier, 2000). Evidence will now be discussed which shows activation in the FFA in response to anthropomorphic rather than human stimuli, which provides further evidence that individuals with ASD may have differentially developed anthropomorphic rather than human expertise.

Research on brain regions such as the fusiform gyrus (FG), which houses the FFA, indicates that the development of facial expertise develops over time. For instance, in children ages five to eight the FG has been shown to be sensitive to objects, but not faces; however, this pattern reverses by the time children reach 11–14 (Scherf et al., 2007). By early to mid-adolescence, the volume of the FG has significantly increased, and this volume is correlated with a person’s ability to recognize and remember faces (Golarai et al., 2007). It is thought that the developed activation of the FG, and in particular the FFA, in response to faces corresponds to an increased necessity to sensitively processing facial information, leading adolescents and adults to become face reading “experts” (Gauthier et al., 2000b). This is significant regarding ToM, as a developed expertise in facial recognition allows for nuanced interpretations when reading emotional expressions (Schmitgen et al., 2016). Individuals with ASD have been shown to demonstrate hypoactivation in the FG and FFA when looking at specifically human faces (Dawson et al., 2005; Humphreys et al., 2008; Pierce and Redcay, 2008). However, the volume of the FG in individuals with ASD is not smaller than NT counterparts, which implies that alternative stimuli may instead activate this region (Whyte et al., 2016).

It may be that FG activity is less impaired, or even intact, in individuals with ASD when social stimuli are anthropomorphic rather than purely human, particularly when stimuli represent a restricted interest. Grelotti et al. (2005) measured FFA activation in a child and adolescent with ASD, one with a heightened interest in the cartoon Digimon, and one without, along with a NT child. During a visual recognition task, participants were shown pictures featuring familiar human faces, unfamiliar human faces, cartoon characters from the show Digimon, and common objects. While the NT participant experienced activation in the FFA only when viewing human faces, the participant with ASD who watched Digimon showed FFA activation only when viewing pictures of Digimon. The participant with ASD without a preference for Digimon showed hypoactivation in the FFA when viewing both faces and Digimon, and instead showed the greatest amount of activation when viewing common objects. This suggests that familiar stimuli related to restricted interests may preferentially recruit the FFA in individuals with ASD, in contrast to human facial stimuli.

Interestingly, research testing ASD participant responses to non-familiar anthropomorphic faces, which may have been, at best, only tangentially related to restricted interests, have also been shown to elicit FFA activation. Jung et al. (2016) measured the neural responses of children with ASD and controls when viewing unfamiliar robot and human faces. Researchers were interested in examining whether robot or human stimuli activated the left hemifield of the brain, where the FFA is located. Results showed that control subjects showed increased activation when gazing at both human and robot faces, indicating activation in the FFA. In contrast, children with ASD only showed left hemifield activity when looking at robot faces and showed hypoactivation in response to human faces.

Whyte et al. (2016), measured FFA activation when adolescents with ASD and controls viewed images of unfamiliar human faces, unfamiliar animal faces (cats and dogs), and common objects. NT participants showed equal activation of the FFA when looking at human and animal faces, in line with research which suggests that in the NT population, human and animal faces are processed similarly (Schirmer et al., 2013). In contrast, those with ASD showed significant hypoactivation when processing human faces. However, those with ASD showed equivalent FFA activation for animal faces, in line with controls, and neither group showed activation when viewing objects. These findings were surprising considering research indicating aberrant gaze behaviors (Guillon et al., 2014) and poor emotional recognition (Gross, 2004) in young children with ASD when viewing both human and animal faces, and activation only in response to common objects when an item is not a specific restricted interest (Grelotti et al., 2005).

All three of these studies may offer support for the role of CIs in ToM for those with ASD, which contends that atypical stimuli may elicit activation in the brain typically reserved for social processing. For instance, the findings produced by Grelotti et al. (2005), which showed FFA activation in response to a preferred cartoon, could be seen as evidence that in ASD the FFA is engaged by restricted interests rather than faces. Similarly, increased FFA activation toward robot faces shown in Jung et al. (2016) may also reflect a heightened response toward a restricted interest, as individuals with ASD have been shown to have a fascination with mechanical systems (Baron-Cohen et al., 2009).

However, FFA activation in response to unfamiliar animal faces, as demonstrated by Whyte et al. (2016) and to a certain extent the unfamiliarity with the robot faces present in Jung et al. (2016), are not as easily explained by CIs. For one, in the Grelotti et al. (2005) study, participants were shown either human faces or whole-body representations of Digimon characters. In contrast, in both Jung et al. (2016) and Whyte et al. (2016) only facial stimuli was visible to participants. Thus, the whole-body details visible to the participant in Grelotti et al. (2005) could have led to increased focus on tertiary aspects of the cartoon that were of restricted interest. The focus on facial stimuli only in Jung et al. (2016) and Whyte et al. (2016), however, limited the ability for participants to focus on aspects of the stimuli that may form a restricted interest category (mechanics, animals) which suggests that activation occurred in response to what were specifically faces. Furthermore, in contrast to one participant’s known interest and familiarity with the Digimon stimuli used by Grelotti et al. (2005), the images used in the other studies were unfamiliar to participants. As evidence suggests that only items relating to specific restricted interests elicit affective neural responses in those with ASD (Cascio et al., 2014), the decreased likelihood that the participants in each of the two study samples possessed a restricted interest underlying their engagement with the animal or robot faces presents an alternative to the CI account.

Together, these studies provide some evidence that individuals with ASD may typically process anthropomorphic rather than human faces, and that the mechanisms underlying this processing be may not be entirely attributable to CIs. This is of interest when forming accounts of ASD, as it suggests that the FFA can be recruited toward general facial processing in this population, particularly when they take a non-human form. This may stem from a possible negative association toward specifically human faces, which has ties to SM. More broadly, these studies also form implications for accounts of anthropomorphism, as it is commonly assumed that anthropomorphism extends from a primarily human representation (Waytz et al., 2010b). In individuals with ASD however, it appears that anthropomorphism occurs in spite of despite a disengagement with human representations. With regard to the third tenet of anthropomorphism by Epley et al. (2007), this may mean that the anthropomorphizing of non-human faces, indicative of facial recognition related FG activity, better elicits agent knowledge in this population. In other words, individuals with ASD increasingly anthropomorphize when agents are human-like and are less inclined to anthropomorphize agents that are strictly human, possibly indicating a closer identification with anthropomorphic creatures.

## Eye Gaze

It is hypothesized that while the holistic processing of faces is a fundamental aspect of facial recognition (Gauthier et al., 2000a), it is the changeable interior aspects of the face may be the most informative of a person’s mental state (Hoffman and Haxby, 2000). Eyes are arguably the most important facial features used for both mental state interpretation (Peterson and Eckstein, 2012) and are particularly implicated in facial recognition (Schyns et al., 2002).

Individuals with ASD have been shown to display marked differences in their attention to eyes compared to NT counterparts, which may be a crucial element of subsequent ToM impairments. For instance, studies have shown that individuals with ASD spend significantly less time attending to eyes when looking at faces (Riby and Hancock, 2009), and attend more to lower regions of the face, such as the mouth (Jones et al., 2008). Both tendencies are often cited as factors leading to their reduced ability to read emotions in eyes (Baron-Cohen et al., 2001; Senju and Johnson, 2009). Researchers such as Tanaka and Sung (2016) have put forth the “eye avoidance” theory of ASD, in which they posit that a lack of eye gaze is due to a heightened emotional arousal in response to eyes. Support for this theory can be found in Kliemann et al. (2012), who showed that individuals with ASD did not simply display an increased fixation toward lower facial elements such as the mouth, but rather an increased avoidance of eyes.

It is also hypothesized that a reduced oxytocin neurohormonal release in response to human co-actors in individuals with ASD (Chaminade et al., 2015a) may make eye contact too sensitizing, as one of the purposes of oxytocin is to reduce anxiety during social engagement (Kosfeld et al., 2005). As research has also found that an administration of oxytocin attenuates neural reactivity when viewing eyes with threatening expressions (Kanat et al., 2015), and promotes eye gaze in individuals with and without ASD (Auyeung et al., 2015), it may be that those with the condition possess weakened neurohormonal priming networks which make eye contact both efficient and rewarding.

Critically, while gazing at human eyes may be uncomfortable for individuals with ASD, as is commonly reported by those with the condition (Grandin and Panek, 2013), this may not extend to anthropomorphic eyes. For instance, Grandgeorge et al. (2016), compared the gaze patterns of NT children and those with ASD when viewing pictures of human, dog, cat and horse faces. While NT children spent more time looking at eyes in general compared to children with ASD, they spent the most time looking at human eyes. In contrast, children with ASD spent the most time looking at the eyes of dogs and cats and spent the least amount of time looking at human eyes. Saitovitch et al. (2013) also produced similar findings. Children were assessed on their eye gaze patterns when looking at movies with cartoon and human characters. While children with ASD looked significantly less at human eyes compared to controls, they, in contrast, spent an equivalent amount of time looking at cartoon eyes. In this way, it may be that while eye gaze never reaches commensurate levels when compared to NT counterparts, eyes may be more salient when they are anthropomorphic.

These findings may provide support for both the SM and CI aspects of ASD. For instance, with regard to CI, both animals and cartoons may pertain to a restricted interest for the individuals with ASD, which would explain longer looking times toward these stimuli. However, as these studies indicate increased attention toward anthropomorphic eyes, in particular, it may be that this type of stimuli does not result in the same degree of emotional dysregulation when returning the gaze and is thus more motivating (SM).

In summary, it appears that individuals with ASD are more likely to anthropomorphize human-like rather than human faces. The three tenets of anthropomorphism outlined in Epley et al. (2007) may support this claim. For one, a need for sociality may cause individuals with ASD to see the social aspects of anthropomorphic characters in typical ways, and this same desire for sociality is not present to the same extent when stimuli are human. Second, it may be that a motivation to fully understand anthropomorphic creatures has led to typical face processing patterns with regard to these stimuli, particularly in studies demonstrating more typical gaze behaviors toward cartoon and animal eyes. As eyes are the most communicative of mental states, it may be that an increased interest in effectance with anthropomorphic stimuli motivates individuals with ASD to gaze at these types of eyes, while an interest in effectance is weakened when an agent is human. Third, it may be that disruptions of self-representations (Lombardo and Baron-Cohen, 2011), have developed into a greater affinity for human-like rather than human stimuli.

The next section will focus on another foundation of ToM processing, the detection, and recognition of biological motion. There is a significant body of research exploring biological motion recognition in ASD, which has largely concluded that from an early age individuals with the condition are not as sensitive to the movements of human agents. As will be discussed, this sensitivity may be intact relative to controls when individuals with ASD view anthropomorphic biological motion, particularly as development progresses.

## Biological Motion Processing

While there are several reasons why anthropomorphic faces may be particularly salient to individuals with ASD, research indicates that anthropomorphic motion may also lead to enhanced social processing. An important element of ToM processing involves the recognition and processing of biological motion (Koster-Hale and Saxe, 2013), which contributes to the perception of sentient animacy, such as the smooth movements of a human as opposed to the jerky, artificial movements of a robot (Freitag et al., 2008). For instance, studies using point-light displays have demonstrated that by only showing several animated points meant to represent limbic movement, individuals are sensitive to points that are analogous with the human body (Johansson, 1973).

One reason that biological motion is salient and informative with regard to ToM is that recognizing it enhances a person’s ability to make predictions about agent behavior (Koster-Hale and Saxe, 2013). For instance, human movements that violate biological laws, such as a finger bending sideways (Costantini et al., 2005), or a human making robotic movements (Saygin et al., 2012), significantly disrupt a person’s ability to predict an agent’s future actions. Thus, sensitivity to biological motion is an important mechanism for ToM processing, as it alerts a person not only to agency but bolsters their ability for social action prediction.

Early in development, infants prefer biological motion over artificial or scrambled motion (Simion et al., 2008), and prefer upright over inverted biological motion (Yoon and Johnson, 2009). By the age of two, they are shown to prefer human over non-human biological motion (Chaminade et al., 2015b). As demonstrated by a person’s ability to infer emotions (Atkinson et al., 2004), dispositions (Brownlow and Dixon, 1997), and intentions (Runeson and Frykholm, 1983) on the basis of biological motion alone, it is conjectured that recognizing mental states may substantially rely on perceptions of another’s motor system honed early in development (Pavlova, 2012).

At a young age children with ASD are shown to be less sensitive to biological motion compared to NTs. For instance, young children with ASD do not differentiate between human and cartoon motion, nor do they prefer artificial or biological motion (Chaminade et al., 2015b). Young children with ASD also struggle to differentiate between biological or scrambled motion when presented in point-light displays (Wang et al., 2015).

Interestingly, research indicates biological motion processing in ASD may be intact later in development when judging non-human biological motion. For instance, Rutherford and Troje (2012), compared adults with ASD to controls on a task using point light displays depicting human, cat and pigeon stimuli. While both groups showed an increased ability to recognize human, then feline, then pigeon motion in a point-light display, there were significant differences between groups in their judgments regarding the direction in which the stimuli were moving. While controls were better able to recognize the direction of human movements, those with ASD were, in fact, better able to determine the spatial direction of the pigeon. This is of particular interest in light of research which indicates that perception of an agent’s spatial direction is analogous to their perceived level of animacy; when an individual struggles to orient to the direction of the stimuli, they are equally diminished in their perceptions of its animacy (Chang and Troje, 2008).

Kaiser and Shiffrar (2012), measured adults with varying degrees of ASD traits on their sensitivity to human, dog, and tractor motion. The magnitude of autistic traits negatively correlated with sensitivity to human motion alone. This suggests that deficits attending to and recognizing biological motion may be specifically impaired with regard to human motion; in contrast, the perception of anthropomorphic motion appears intact.

Both SM and CIs patterns in ASD may be responsible for an insensitivity to human biological motion, and a possibly intact sensitivity to anthropomorphic biological motion. For instance, the propensity for NT individuals to “see human,” which underscores a sensitivity to human biological motion, may be indicative of increased neural reward activation when processing human movement. Individuals with ASD, who experience hypoactivation in reward systems when interacting with human stimuli (Chaminade et al., 2015a), may, therefore, be less primed to attend to human biological motion. Indeed, research asking participants with different degrees of ASD related traits to assign a value to forms with varying degrees of biological motion found that those with a higher degree of ASD related traits did not assign greater value to human biological motion (Williams and Cross, 2018). This may speak to a decreased motivation to closely attend or engage with purely human stimuli or, equally, an enhanced interest in human-like or anthropomorphic agents.

With regard to the CIs in ASD, it may also be that the motion of animate, non-human creatures, represent motion which is more in line with restricted interests. For instance, individuals with ASD often show restricted interest in objects with mechanical movements (Turner-Brown et al., 2011). This may underlie individuals with ASD’s atypical attribution of “humanness” to non-biological, mechanical motion observed in androids (Kumazaki et al., 2017), which in NT’s is viewed as less salient and significantly disrupts action perception (Saygin et al., 2012). In this way, individuals with ASD may be both less sensitive to anomalies in human motion as they are less primed to process it preferentially (SM), and the atypicality of non-biological motion, which NTs find unnatural, are of heightened interest to individuals with ASD (CI).

In summary, an important aspect of ToM is the recognition of biological motion, which indicates that the bodily movements of an agent are indicative of human action. Recognizing motion as indicative of human movement allows an individual to better form predictions regarding that agent’s intentions and goal-directed behaviors. Beginning at an early age, NT infants are sensitive to human biological motion. Research has found a different developmental trajectory in young children with ASD, who do not show a preference for either biological motion or human agency. This possibly extends throughout adulthood, though some research indicates that by adulthood individuals with ASD are better able to recognize human motion in line with NT adults, though there is some evidence that human biological motion recognition continues to be impaired (Kaiser and Pelphrey, 2012).

Interestingly, two studies indicate that biological motion detection and judgments regarding the direction of biological motion is not impaired in relation to animal motion; individuals with ASD related traits have been shown to be impaired only when attending to human not dog biological motion (Kaiser and Shiffrar, 2012), and those with ASD are best able to predict the direction of pigeon rather than human motion, to an even larger degree than controls (Chang and Troje, 2008). In this way, the processing and recognition of specifically human biological motion may be impaired, while perceptions of anthropomorphic motion may be intact. This may mean that individuals with ASD have developed a sensitivity for non-human motion in line with CI, and are less interested in human biological motion in line with SM.

The finding that biological motion is enhanced when individuals with ASD view anthropomorphic stimuli may also correspond to the Epley et al. (2007) model of anthropomorphism in a similar fashion as findings on anthropomorphic face processing. In particular, an increased ability to anthropomorphize anthropomorphic versus human biological motion may indicate an enhanced social response toward anthropomorphic creatures, in line with the first tenet of sociality. In line with the second tenet of enhanced effectance, if animals represent a restricted interest, individuals with ASD may display a heightened interest in processing anthropomorphic stimuli efficiently, and are thus primed to detect anthropomorphic biological motion. The last of the Epley et al. (2007) tenets, which states that anthropomorphism occurs through eliciting agent knowledge, may be particularly at play in the processing of anthropomorphic biological motion in ASD. For instance, research indicates that the recognition of biological motion is enhanced when an individual is able to map physical aspects of animal motion through the use of a corresponding human reference (Welsh et al., 2014), such as relating the bipedal motion of a walking pigeon to that of a walking human figure. As a physical sensing of the self has been shown to be impaired in those with ASD (Lombardo et al., 2010) it may be that a diminished sense of personal motion may lead to a greater insensitivity to human motion, while not diminishing a sensitivity to anthropomorphic motion. Indeed, if individuals with ASD are more attune to animal rather than human stimuli, as research suggests (Celani, 2002; Prothmann et al., 2009), it may be that elicited agent knowledge in this population takes a more anthropomorphic rather than human form. In the next section, findings relating to increased engagement with anthropomorphic stimuli in individuals with ASD and how this related to ToM is discussed.

## Increased Engagement With Anthropomorphic Stimuli and Theory of Mind

It is suggested throughout this review that, be it facial processing or recognition of biological motion, individuals must experience some type interest in a stimulus in order to process it socially. The role of social engagement in anthropomorphism is also central to the Epley et al. (2007) model, in which a desire for sociality is cited as the most important determinant of anthropomorphism. Thus, an underlying argument in this review is that individuals with ASD may find anthropomorphic stimuli more socially motivating than human stimuli, which underlies their enhanced social processing of such stimuli.

Silva et al. (2015) directly tested individuals with ASD on their broader engagement with anthropomorphic stimuli. Adolescents and adults with ASD and age-matched controls were tested on their reaction times when performing the Approach-Avoidance Task (Rinck and Becker, 2007). In this task, the participants’ approach or avoidance of either cartoon or human photographed images were measured by the speed in which they manipulated pictures of emotionally positive, negative and neutral social scenes through either the pushing (minimizing image) or pulling (enlarging) of a joystick. Results showed that unlike NTs, those with ASD were significantly more avoidant of emotionally positive photographs, and in contrast found emotionally positive cartoons significantly more approachable. Thus, it may be that the heightened anthropomorphism seen in this population toward anthropomorphic stimuli is reflective of a desire for sociality, a need which may not be met within traditional human encounters.

In a study that more closely examined anthropomorphic engagement and it’s effect on ToM, NT and ASD adolescents were tested on their ability to recognize emotional expressions in three types of media (still images, dynamic images, and auditory noise) across human and cartoon stimuli (Brosnan et al., 2015). Results showed that NT adolescents were superior to those with ASD in emotion recognition of human stimuli across all three modalities. This, however, did not extend to animated (cartoon) stimuli. In fact, not only did individuals with ASD significantly improve within group scores on emotion recognition when viewing cartoon versus human stimuli, they outperformed controls in the recognition of static cartoon stimuli. However, it is important to note that accuracy for animated stimuli in the ASD group was never as high as accuracy for human stimuli in the NT group, indicating that cartoon presentation does not entirely compensate for relative ToM-related deficits.

One finding of particular interest related to differences in processing strategies between groups. The researchers found that in the control group, emotion recognition for cartoon and human stimuli were correlated, meaning that the strategies used by controls in one modality were similarly utilized in others. However, no such correlations were found within the ASD group. This indicates that the manner in which individuals with ASD were processing cartoon stimuli was not employed when processing human images, a possible indication that cartoon stimuli were viewed as “special” while human stimuli were not.

The above research suggests that engagement and motivation with regard to anthropomorphic stimuli could ameliorate ToM deficits for those with ASD. One study that tested this was conducted by Golan et al. (2010), and explored whether improving ToM by anthropomorphizing non-human agents could lead to transferable gains in human ToM. In this study, children with ASD aged 4–7 engaged in a 4-week intervention in which they watched instructional ToM videos acted out by toy vehicles grafted with real faces. Following the intervention, the children were assessed in relation to two control groups, one with ASD and one without ASD, both of whom did not partake in the intervention on their ability to generalize learned facial expressions and utilize emotional vocabulary. Results indicated that while the experimental group was indistinguishable from the control ASD group at pre-test, by post-test they had improved to the level of the control group on all four measures. Central to these findings was the children’s demonstrated ability to generalize content to not only novel anthropomorphic stimuli but novel human stimuli. This indicates that the intrinsic interest individuals with ASD showed toward areas of restricted interest may have promoted their interest and understanding in human stimuli.

In relation to the Epley et al. (2007) model, anthropomorphic stimuli may enhance sociality, increase the desire for effectance, and is not viewed as incongruent with the physical self. The following section will focus on the second tenet of the model, in which it is found that a desire for efficacy promotes anthropomorphism. Studies documenting an increased desire for efficacy in individuals with ASD when processing anthropomorphic characters due to stylization/exaggeration, and extensive previous experiences with such stimuli, will be explored.

## Effectance With Stylization/Exaggeration

As previously discussed, research indicates that individuals anthropomorphize in order to increase their efficacy in understanding a non-human entity and this is enhanced when behavior is less predictable (Waytz et al., 2010b). One aspect of anthropomorphic stimuli that may particularly increase effectance of individuals with ASD is the stylization and exaggeration of social features in such agents, which may highlight a sense of unpredictability regarding their intentions. Support for this comes from research showing that within this population the recognition of changes in emotion may be impaired, while the perception of changes in motion is intact (Han et al., 2015). This may mean that the exaggerated movements used by anthropomorphic characters to express emotions may be more noticeable to those with the condition, while changes in emotion may be missed and thus not utilized when making judgments of unpredictability.

Research on animal movement, for instance, indicates that individuals largely rely on physical movements, such as the motion of the tail and muzzle cues like the baring teeth, when identifying an animal’s mental state (Tami and Gallagher, 2009). Thus individuals with ASD may be better equipped to attend to animal emotion, as it involves the interpretation of overt movement rather than subtle changes in facial expression. In this way, the unpredictability of animal agents may be more noticeable, thus leading to a greater desire for effectance. Cartoon characters are also characterized by exaggerated motion (Thomas et al., 1995), which serves to direct attention toward socially relevant aspects of the animation (Gielniak and Thomaz, 2012). In a similar way to animal agents, individuals with ASD may be more primed to attend to the unpredictability of cartoon motion as it is exaggerated and thus more salient.

Carter et al. (2016) provides preliminary support for the hypothesis that exaggerated motion in anthropomorphic stimuli increases interest in effectance. In this study, children with ASD interacted with animated avatars with varying degrees of facial emotional exaggeration. When an avatar showed exaggerated facial motion, compared to dampened or realistic motion, nonverbal behaviors such as gaze or gesturing significantly increased. This is in line with research showing that individuals with ASD are less impaired when interpreting overt emotional expressions, and struggle more with the detection of subtle facial emotional changes (Rump et al., 2009). Anthropomorphic faces, which exaggeration makes more emotionally intense (Hyde et al., 2014), may heighten their unpredictability and lead to a greater desire for effectance, while subtle changes in realistic human agents are less salient, and result in a decreased desire for effectance.

## Effectance From Cartoon and Animal Experience

### Cartoon Experience

An important aspect of the desire for effectance brought up in Epley et al. (2007) is people anthropomorphize out of a desire for ‘closure’ or understanding of an agent. One reason that individuals with ASD may anthropomorphize cartoon stimuli more than human stimuli is that familiarity with such content has led to an increased sense of self-efficacy in understanding such stimuli. Heightened interest and time spent attending to animated stimuli is well documented in this population. For instance, survey data shows that adolescents with ASD spend a significant amount of time engaging with electronic screen media (Mazurek et al., 2012). Surveys given to parents of children with ASD indicate that electronic screen engagement is their most common leisure activity, in particular animated television shows and movies (Shane and Albert, 2008). Kuo et al. (2014) also found that within a sample of adolescents with ASD, cartoon television programs were the most popular television genre, and 66% of the sample reported a preference for animation over any other type of media.

Drawing a causal relation between cartoon viewing and increased ToM abilities with regard to cartoon stimuli remains ambiguous. As has been discussed previously in this review, there are reasons why the stylized exaggeration inherent to animated media may attract individuals with ASD to this medium. For one, the exaggerated and amplified motion may allow for greater success when making ToM judgments, leading to enhanced self-efficacy and thus greater enjoyment of this type of media. As individuals with ASD report increased familiarity and exposure to this form of media, it may be that they have an increased expertise in processing cartoon stimuli, which has led to the type of FFA activation that enhances ToM related processing. This may increase a desire for effectance in relation to cartoons, as individuals with ASD may feel better equipped to understand the meaning behind the social acts depicted in cartoon form due to their increased exposure, thus increasing their tendency to anthropomorphize (Epley et al., 2007).

### Animal Experience

Individuals with ASD also show increased motivation and experience regarding animal stimuli. For instance, Celani (2002) compared children with ASD to NTs, and those with intellectual disabilities, on their preferences for human, animal and object stimuli. While children with ASD significantly preferred objects over human stimuli, they showed a significantly greater preference for animals than all other types of stimuli. Prothmann et al. (2009) showed children with ASD interacted significantly more frequently and for a longer duration with a dog than a person or toy, when through a free-choice paradigm. Both provide evidence of an implicit preference in individuals with ASD for animal stimuli, which may motivate attention to animals over humans.

With regard to animal experience, it is estimated that 1 in 4 children with ASD have participated in animal therapy at some point, and two-thirds of parents report improvements following animal-assisted interventions (Christon et al., 2010). Research also indicates that families of children with ASD may have a particularly high rate of pet ownership, as 81% of families with a child with ASD surveyed on pet ownership reported owning pets (Carlisle, 2014), while the national average is around 66% according to the American Veterinary Medical Association [AVMA] (2012). Further findings in this study indicated that 94% of children with ASD were described by parents as having bonded with their pets, with common bonding activities including talking and actively playing and petting their pets. Parents commonly reported that they believed pets provided specific benefits to their children with regard to alleviating common challenges related to ASD, and 26% of parents reported that the perceived benefits of animal contact on ASD symptoms factored into their decision to own a pet, particularly dogs. Surveys of individuals with ASD also indicates strong perceived attachments between themselves and their pets (Carlisle, 2015).

Together, these results indicate that not only do individuals with ASD commonly have extensive contact with animals but that these encounters are viewed quite positively by both themselves and close others. Given that individuals with ASD often report a significantly high degree of negative social experiences (White et al., 2011; Lamport and Turner, 2014), and decreased social self-efficacy (Vickerstaff et al., 2007), successful encounters with animals may increase a desire for effectance, as previous positive encounters with animals may have incentivized understanding animal agents (Epley et al., 2007).

Considering this evidence, it appears that individuals with ASD on average tend to have frequent and positive experiences interacting with animals and cartoons, either through media engagement, structured animal-assisted interventions, pet-ownership, or all three. In this way, the positive social experiences individuals with ASD have had with regard to anthropomorphic agents may lead to greater motivation to interact effectively with such stimuli. As individuals with ASD have experienced social reward associated in particular with animal engagement, anthropomorphizing animals may happen out of a desire to further understand and predict the behavior of this stimuli. Additionally, a heightened exposure to cartoons may lead individuals with ASD to view understanding the mental states of cartoons as within their control. In contrast, it may be that individuals with ASD view the processing of human stimuli as less in their control, and they show decreased anthropomorphism for human agents.

## Summary

The processing of mental states is a complex, multi-faceted procedure that requires lower-level inputs in order to produce higher-order ToM explanations. Individuals with ASD have been shown to struggle with ToM throughout development, and evidence suggests that lower-level processing impairments such as reduced facial and biological motion processing may play a significant role in this disruption. In particular, it appears that individuals with ASD have early insensitivities to human agency, namely attending to human faces and human biological motion.

While evidence suggests that individuals with ASD show significant deficits in relation to recognizing and processing human stimuli, they are conversely shown to display a heightened interest in non-social stimuli compared to NTs. The SM and CI aspects of ASD complement one another in their explanations of these deficits. In relation to SM, early deficits in relation to human social processing, which primes NTs to preferentially attend to such stimuli through an associated neural reward system, is impaired in those with ASD. This may lead to decreased reward circuitry, and thus less holistic and preferential processing of human stimuli, which impairs ToM processing at lower levels of input. Additionally, the preference individuals with ASD show toward non-social stimuli (CI), particularly objects in the environment that have ordered motion or systems, may reflect a preference to attend to items of restricted interests in place of social stimuli. In this way, the increased motivation to attend to non-social stimuli may impact the motivation to attend to less-ordered, more complex social stimuli.

However, the many studies detailed in this review indicate that engagement with anthropomorphic stimuli may function as a bridge for individuals with ASD to attend to social stimuli. In line with SM, it is hypothesized that the developed stressors associated with human contact may not extend to human-like stimuli. In this way, individuals with ASD may be more motivated to attend to anthropomorphic stimuli in typical ways, as anthropomorphic stimuli feature properties that differentiate them from purely human agents. It is also hypothesized that as individuals with ASD are able to attend to motion, and struggle with the nuances of emotion, an ability to decode animal and cartoon emotion using overt movement cues could make social processing less difficult, thereby enhancing SM. The frequent exposure to cartoons and animal agents may also serve to enhance motivational engagement with such stimuli.

Also playing an important role in anthropomorphic social processing is found in aspects of CIs in those with ASD. Properties of anthropomorphic agents that correspond to restricted interests, including stylized physical properties and an association with an exaggerated motion, may direct attention to these agents over and above agents that are purely human. For instance, individuals with ASD report an enhanced interest and experience with cartoon stimuli, and the overt, exaggerated aspects of cartoon motion may be particularly salient. In this way, anthropomorphic agents may represent an area of expertise for individuals with ASD, therefore enhancing their ability to attend to them holistically.

For these reasons, it is suggested that while the social processing of human stimuli appears to be impaired in this population, the processing of anthropomorphic stimuli is either less pronounced, intact or even enhanced. Thus, using anthropomorphic stimuli to develop social processing in individuals with ASD may help ameliorate a decreased motivation to engage with human stimuli. It may also aid individuals with ASD in the processing of social over non-social stimuli, as anthropomorphic creatures are social agents, yet they also possess physical characteristics reminiscent of restricted, non-social interests. The implications of these findings are discussed below.

## Implications

There are several important implications for the increased social processing of anthropomorphic stimuli in individuals with ASD. Chief among them is the possibility that increasing social cognitive development in relation to anthropomorphic stimuli may serve as a scaffold for transferring these skills to human stimuli. There is some evidence that supports this claim. For instance, Golan et al. (2010) showed that improvements understanding mental state language in connection to anthropomorphic characters transferred to social gains with human stimuli. This indicates that the use of areas of CI when combined with human elements may help improve ToM when interacting with non-CI related agents.

Research on animal-assisted interventions such as equine therapy indicates that skills learned with animal agents transferred to real life social improvements even when measured 1-month post-trial (Gabriels et al., 2015). Studies measuring naturalistic social improvements also show that in the presence of animals, real-life social functioning can improve, and importantly lead to greater peer acceptance (O’Haire et al., 2013). These studies indicate that the enhanced social processing, and the motivation experienced by individuals in relation to anthropomorphic stimuli, may transfer to improvements in human interactions.

Perhaps most significant is the possibility that perceived self-efficacy with anthropomorphic stimuli can lead to gains in perceived self-efficacy in relation to humans, and human encounters. Underlying the “eye avoidance” hypothesis of ASD (Tanaka and Sung, 2016) is that individuals with ASD develop gaze aversion in relation to human contact, as they may implicitly equate eye gaze with social demands that they cannot meet. For instance, evidence shows that in preschool there is not the same aversion to mutual gaze and emotional dysregulation in children with ASD (Nuske et al., 2015), and 2-year-old children with ASD show eye indifference rather than eye avoidance, as they can be primed to view eyes (Moriuchi et al., 2016).

However, research also indicates that in adults with ASD there is a distinct aversion to direct eye-gaze (Kliemann et al., 2012), and that direct eye gaze results in hyperactivation in subcortical areas of the brain, indicating dysregulation (Hadjikhani et al., 2017). This may indicate that early eye indifference later results in eye avoidance, leading to a possibility that commensurate with age, individuals with ASD may develop a human-specific social aversion. In contrast, early eye insensitivity may not impact individuals with ASD’s perceived self-efficacy with anthropomorphic agents. In this way, the negative associations that may impede further development of social processing in relation to human stimuli may not interfere with development in regard to anthropomorphic social processing. This reflects theories of ASD relating to social compensation (Livingston and Happé, 2017), and it may be that the difficulties associated with human agents are compensated for when interacting with non-human agents.

With regard to compensation, it may be that an ability to process anthropomorphic social cues creates a pathway to developing social processing competencies, and this may be a bridge to developing competencies with human stimuli. For instance, research indicates that the same brain regions are recruited when individuals use ToM in relation to animals as they do in relation to humans (Desmet et al., 2017), and those facial expressions in both animals and humans are processed similarly (Schirmer et al., 2013). Interestingly, research indicates that when assessing the emotions of dogs, individuals often used their own emotions as a template (Konok et al., 2015). In this way, engagement with mentalizing about animals may lead to increased processing of personal emotions, which has been shown to be impaired in individuals with ASD (Jackson et al., 2012), and thus may be an important mechanism for ToM improvement (Allan et al., 2017). Effective reasoning about anthropomorphic social agents may, therefore, transfer to efficacy with human agents and even efficacy in understanding the self.

There are several implications for interventions with regard to enhanced social processing for anthropomorphism. One is that, in line with Golan et al. (2010), it may be advantageous to use anthropomorphic stimuli when engaging individuals with ASD in ToM interventions. In particular, future interventions of this nature should focus on scaffolding, and slowly applying strategies toward more human-like stimuli presentations.It is also of interest to examine how longitudinal interventions with anthropomorphic stimuli may differentially affect what may be a developed aversion to human stimuli in older individuals with ASD.

In particular, O’Haire et al. (2013) indicates that interactions with animals by both children with ASD and NTs in a classroom setting enhances social reciprocity. It may be that structuring inclusive classroom settings to involve animal contact may improve social outcomes for individuals with ASD and foster greater peer acceptance. This may help counteract some of the negative social experiences often reported by individuals with ASD, and lead to greater self-efficacy in relation to social encounters. Experiential accounts from individuals with ASD often report attachment and elevated self-esteem in relation to anthropomorphic agents, particularly animals. It may be that anthropomorphism for this population allows those with ASD to experience social engagement in a way that feels more natural, and thus can aid in transferable ToM gains to other social settings.

In closing, the Epley et al. (2007) model of anthropomorphism uses three tenets to explain why people anthropomorphize. It is suggested that individuals with ASD may use anthropomorphic creatures as a social outlet of sorts, and in this way, a desire to see the social aspects of anthropomorphic creatures leads to better holistic processing of this stimuli. Individuals with ASD may also have a greater desire to understand anthropomorphic creatures, as they have had success understanding and interacting with such agents, and the agents have properties related to CIs, which enhances a desire for effectance. Additionally, a decreased salience for humans and an increased salience for anthropomorphic characters, perhaps tied to exaggerated motion and a poor detection of emotion, may lead to a stronger recognition of unpredictability, thus enhancing a desire for effectance with anthropomorphic creatures. Finally, individuals with ASD have a diminished physical sense of self and are less sensitive to anomalies in the human form. While this impedes anthropomorphizing non-human creatures in those who are NT, this may not lead to the same types of processing deficits in individuals with ASD. Conversely, the aspects of the physical self that, in individuals with ASD, are less salient or noticeable, may lead to a heightened identification with other “more than human” and thus more exaggerated stimuli.

At present, investigations into anthropomorphism have found that ToM impairments correspond to impairments anthropomorphizing (Cullen et al., 2014). It may be of interest to examine whether this is unilaterally the case with individuals with ASD. For instance, research shows that in anthropomorphic assessments using animated shapes, individuals with ASD are less able to anthropomorphize (Abell et al., 2000). However, it may be that with more socially enriched stimuli, such as animal or human cartoon stimuli, individuals with ASD may display a different pattern with regard to anthropomorphism and ToM. Additionally, as is explored by Brosnan et al. (2015), deficits relating to ToM may be ameliorated when stimuli take a less human form. It would be of particular interest to test whether this can be replicated, particularly through the use of non-visual ToM paradigms, in order to assess the purely cognitive aspects of mental state representations and their connection with anthropomorphism in this population. It would also be of interest to further understand how anthropomorphism and self-perceptions interact in ASD, and whether anthropomorphism can serve as a pathway for improving intrapersonal as well as interpersonal social processing, and ToM more generally.

In conclusion we have highlighted how the ability to anthropomorphize may not only be intact in those with ASD, but those with the condition may even display a particular affinity for seeing human in the non-human. Evidence suggests that ToM abilities, which are usually disrupted in this population, may be ameliorated, spared, or even enhanced when they are directed toward anthropomorphic rather than human agents. As we have shown, anthropomorphizing may be a potential scaffold for improving ToM abilities more generally in this population, as they correspond with a number of strengths intrinsic to ASD. Identifying and capitalizing on such strengths may be the key to improving ToM, and allowing those with ASD better integration within the wider social world.

I moved full circle form being a wild thing out of context as a child, to being a wild thing in context with a family of gorillas, who taught me how to be civilized. They taught me the beauty of being wild and gentle together as one (Prince-Hughes, 2004, p. 1).

## Author Contributions

GA and LC contributed to the design, structure, content of the review, and writing the final draft. GA prepared the first draft.

## Conflict of Interest Statement

The authors declare that the research was conducted in the absence of any commercial or financial relationships that could be construed as a potential conflict of interest.
